# Multidimensional structural analyses revealed a correlation between thalamic atrophy and white matter degeneration in idiopathic dystonia

**DOI:** 10.1093/braincomms/fcaf026

**Published:** 2025-01-20

**Authors:** Jinping Xu, Qinxiu Cheng, Yue Zhang, Yuhan Luo, Linchang Zhong, Huiming Liu, Haoran Zhang, Zhengkun Yang, Jiana Zhang, Zilin Ou, Zhicong Yan, Kangqiang Peng, Gang Liu

**Affiliations:** Institute of Biomedical and Health Engineering, Shenzhen Institutes of Advanced Technology, Chinese Academy of Sciences, Shenzhen 518055, China; Institute of Biomedical and Health Engineering, Shenzhen Institutes of Advanced Technology, Chinese Academy of Sciences, Shenzhen 518055, China; Department of Neurology, The First Affiliated Hospital, Sun Yat-sen University, Guangdong Provincial Key Laboratory of Diagnosis and Treatment of Major Neurological Diseases, National Key Clinical Department and Key Discipline of Neurology, Guangzhou 510080, China; Department of Neurology, The First Affiliated Hospital, Sun Yat-sen University, Guangdong Provincial Key Laboratory of Diagnosis and Treatment of Major Neurological Diseases, National Key Clinical Department and Key Discipline of Neurology, Guangzhou 510080, China; Department of Medical Imaging, Sun Yat-Sen University Cancer Center, State Key Laboratory of Oncology in Southern China, Collaborative Innovation Center for Cancer Medicine, Guangzhou 510060, China; Department of Medical Imaging, Sun Yat-Sen University Cancer Center, State Key Laboratory of Oncology in Southern China, Collaborative Innovation Center for Cancer Medicine, Guangzhou 510060, China; Institute of Biomedical and Health Engineering, Shenzhen Institutes of Advanced Technology, Chinese Academy of Sciences, Shenzhen 518055, China; Department of Neurology, The First Affiliated Hospital, Sun Yat-sen University, Guangdong Provincial Key Laboratory of Diagnosis and Treatment of Major Neurological Diseases, National Key Clinical Department and Key Discipline of Neurology, Guangzhou 510080, China; Department of Neurology, The First Affiliated Hospital, Sun Yat-sen University, Guangdong Provincial Key Laboratory of Diagnosis and Treatment of Major Neurological Diseases, National Key Clinical Department and Key Discipline of Neurology, Guangzhou 510080, China; Department of Neurology, The First Affiliated Hospital, Sun Yat-sen University, Guangdong Provincial Key Laboratory of Diagnosis and Treatment of Major Neurological Diseases, National Key Clinical Department and Key Discipline of Neurology, Guangzhou 510080, China; Department of Neurology, The First Affiliated Hospital, Sun Yat-sen University, Guangdong Provincial Key Laboratory of Diagnosis and Treatment of Major Neurological Diseases, National Key Clinical Department and Key Discipline of Neurology, Guangzhou 510080, China; Department of Medical Imaging, Sun Yat-Sen University Cancer Center, State Key Laboratory of Oncology in Southern China, Collaborative Innovation Center for Cancer Medicine, Guangzhou 510060, China; Department of Neurology, The First Affiliated Hospital, Sun Yat-sen University, Guangdong Provincial Key Laboratory of Diagnosis and Treatment of Major Neurological Diseases, National Key Clinical Department and Key Discipline of Neurology, Guangzhou 510080, China

**Keywords:** idiopathic dystonia, voxel-based analyses, voxel-based morphology, fixel-based analyses, surface-based morphometry

## Abstract

Although aberrant changes in grey and white matter are core features of idiopathic dystonia, few studies have explored the correlation between grey and white matter changes in this disease. This study aimed to investigate the coupling correlation between morphological and microstructural alterations in patients with idiopathic dystonia. Structural T1 imaging and diffusion tensor imaging were performed on a relatively large cohort of patients. Multidimensional structural analyses, including voxel-based analyses, voxel-based morphology, fixel-based analyses and surface-based morphometry, were performed to explore these structural alterations. Probabilistic tractography and correlation analyses were employed to examine these relationships. A total of 147 patients with idiopathic dystonia and 137 healthy controls were recruited in this study. There were no significant differences in the cortical morphometry between patients with idiopathic dystonia and healthy controls using voxel- and surface-based morphometry. However, the grey matter volume of the bilateral thalamus, fractional anisotropy in the right anterior corona radiata, right retrolenticular part of the internal capsule and right posterior corona radiata, and the fibre density and cross-section combined in the fibre tract connecting the left ventral posterolateral thalamic nucleus and left area 5 m, were significantly decreased in patients with idiopathic dystonia compared with those in healthy controls. Furthermore, the reduced grey matter volume in the right thalamus not only correlated with the disease duration but also with the reduced fractional anisotropy in the right posterior corona radiata and decreased the fibre density and cross-section combined in the fibre tract connecting the left ventral posterolateral thalamic nucleus and the left area 5 m in patients with idiopathic dystonia. These findings suggest that the thalamus is structurally impaired in idiopathic dystonia and that microstructural disruption in thalamocortical projections occurs secondary to thalamic atrophy.

## Teaser

Xu *et al*. aimed to investigate the correlation between grey matter morphological changes and white matter microstructural alterations in a relatively large cohort of patients, highlighted the involvement of thalamus as a central region in the pathophysiology of dystonia, and suggested a potential cascade of microstructural disruptions in thalamocortical pathways.

## Graphical abstract

**Graphical abstract:**
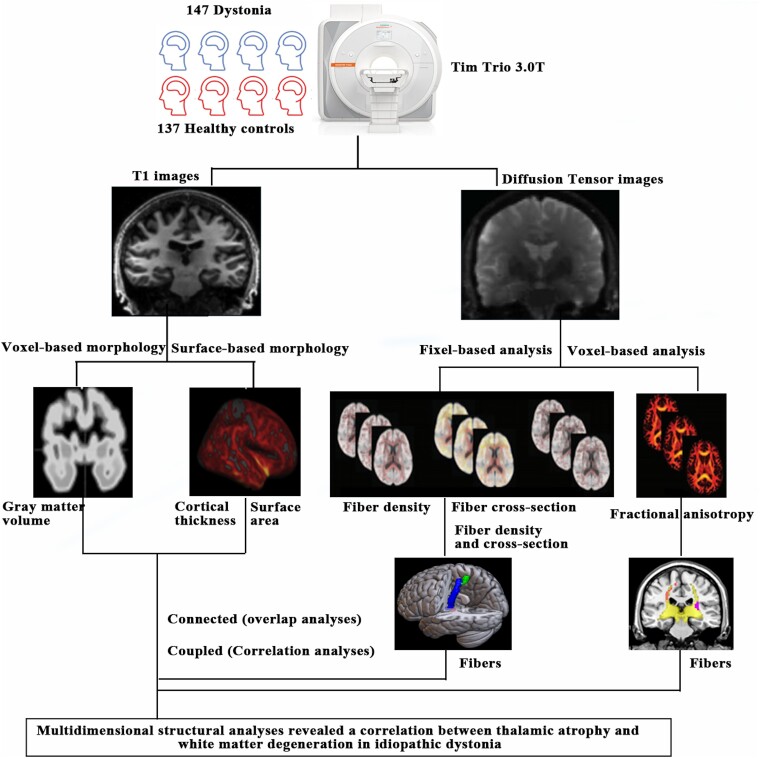

## Introduction

Idiopathic dystonia is a hyperkinetic movement disorder characterized by repetitive movements and/or abnormal postures in various body regions due to involuntary sustained or intermittent muscle contractions.^1^ The most prevalent forms of adult-onset dystonia are cervical dystonia and blepharospasm.^2^ Despite considerable efforts to identify its aetiology, the complete pathophysiology of dystonia remains poorly understood. A substantial body of evidence suggests that basal ganglia dysfunction may play a key role in its pathophysiology.^3,4^ However, recent human neuroimaging studies have contributed to understanding the anatomical basis of dystonia and have detected abnormalities not only in the basal ganglia but also in various cortical regions, the cerebellum, and the brainstem, as well as their connections.^5^ Based on this information, a network model for the pathogenesis of dystonia, which mainly includes the basal ganglia-thalamocortical and cerebello-thalamocortical motor circuits,^6^ has been proposed in recent years. Despite a high heterogeneity of clinical phenotypes in adult-onset idiopathic dystonia, the different forms of dystonia possibly share a common neuroanatomical origin.^7,8^ However, current neuroimaging studies investigating grey or white matter abnormalities have failed to reveal a common pattern of changes in patients with idiopathic dystonia.^9-16^ Discrepancies across studies may result from factors such as differences in treatment regimens or data analysis.^17^ More importantly, the relatively small number of patients recruited in these studies may be a significant limitation, leading to discrepancies. In addition, the other key issue is the cause or consequence of interpreting grey or white matter changes in idiopathic dystonia. This involves understanding the coupling correlation between the morphological and microstructural alterations in idiopathic dystonia. Therefore, a thorough understanding of the disease mechanisms can inform future research and contribute to the development of new treatment strategies. Consequently, addressing the questions identified in this study is vital for advancing our knowledge of idiopathic dystonia and its clinical implications. Currently, in vivo, high-resolution neuroimaging and diffusion-weighted magnetic resonance imaging (MRI) sequences have provided new perspectives and deeper insights into the neuro-mechanisms underlying idiopathic dystonia. Brain morphometric parameters for grey matter are usually calculated using voxel-based morphometry (VBM), which measures the quantity of tissue within a voxel and is dependent on local cortical thickness (CT) and/or cortical surface area (SA).^18^ In addition, surface-based morphometry (SBM) estimates cortical SA and thickness, showing high sensitivity in detecting cortical changes, especially those associated with gene mutations.^19^ Most knowledge about white matter organization comes from diffusion tensor imaging (DTI), which examines axonal organization using parameters such as fractional anisotropy (FA) to estimate the directional coherence of diffusion within a voxel.^20^ Recently, a new method termed fixel-based analysis (FBA) has been proposed to explore the morphometric properties of fibre pathways.^21^ FBA employs a spherical harmonic representation of diffusion, allowing for better representation of complex multi-fibre geometry compared with tensor-based models. This enables the separation of anatomically informative metrics for distinct fibre populations within a voxel.^22^ Three major parameters used in FBA include the fibre cross-section (FC), the density of the fibres (FD) with a fixel and the fibre density and cross-section (FDC) for estimating the total number of fibres in a fibre bundle. As these structural parameters are designed and optimized to capture different structural properties of brain tissues, brain morphometry and brain microstructure, it is ideal to combine this multidimensional information to create a comprehensive map revealing morphological and microstructural alterations and their correlation in idiopathic dystonia.

In this study, we collected structural T1 images and DTI data from patients with idiopathic dystonia. Whole-brain multidimensional structural analyses were performed to explore macro- and microstructural alterations in patients with idiopathic dystonia. Probabilistic tractography and correlation analyses were performed to examine the correlation between morphological and microstructural alterations in patients with idiopathic dystonia.

## Materials and methods

### Standard protocol approvals, registrations and patient consents

This study was approved by the Ethics Committee of the First Affiliated Hospital of Sun Yat-sen University [(2020)323]. Written informed consent was obtained from all participants according to the Declaration of Helsinki.

### Participants

Patients were recruited from the outpatient clinic for movement disorders and were diagnosed with adult-onset blepharospasm, blepharospasm-oromandibular dystonia, or cervical dystonia following the published criteria by two senior neurologists (G Liu and ZL Ou).^23-25^ The patients were excluded if they met the following criteria: (i) age < 18 years; (ii) had received botulinum toxin injections within 3 months before imaging; (iii) showed evidence of traumatic brain injury, stroke, Alzheimer’s disease, Parkinson’s disease, or epilepsy; (iv) had a history of medication exposure before the onset of dystonia or a family history of movement disorders; (v) had medical implants contraindicated for undergoing cerebral MRI. Finally, 147 idiopathic dystonia cases (74 with blepharospasm, 31 with blepharospasm-oromandibular dystonia and 42 with cervical dystonia) and 137 healthy controls were continuously included in this study (Table 1). Some patients were taking different types of medications, including anticholinergic drugs, benzodiazepines, muscle relaxants, antidopaminergic drugs, antiepileptic drugs and anti-anxiety drugs. However, none of the patients had taken any of these medications for at least 24 h before the MRI scan.^26^

**Table 1:** Subjects’ demographic and clinical characteristics

Groups	Idiopathic dystonia (*n* = 147)	Healthy controls (*n* = 137)
Blepharospasm	74	
Blepharospasm-oromandibular dystonia	31	
Cervical dystonia	42	
Sex (female/male)	57/90	49/88
Age, years	49.52 ± 12.16	49.78 ± 12.60
eTIV, mm^3^	1416095 ± 185554	1 394175 ± 189900
Disease duration, years	3.66 ± 3.96	
BoNT injections, yes/no	85/62	
BoNT injections duration, years	1.21 ± 2.26	
BFMDRS-M score	6.90 ± 2.63	
BFMDRS-D score	4.45 ± 2.11	

Values shown as mean ± standard deviations.

BFMDRS-D/M, disability/motor section of the Burke–Fahn–Marsden Dystonia Rating Scale; BoNT, botulinum toxins; eTIV, estimated total intracranial volume.

### Clinical assessment

Demographic and clinical characteristics, including patients’ age, sex, disease duration, botulinum toxin injections duration and number of botulinum toxin injections, were collected from all patients through face-to-face interviews before the MRI scans. Disease severity was assessed immediately before MRI using the Burke–Fahn–Marsden Dystonia Rating Scale (BFMDRS)^27^ in patients. The BFMDRS comprises two components: the motor severity scale and the disability scale. The motor severity scale evaluates nine body regions, including the eyes, mouth and jaw, speech and swallowing, neck, arms, trunk and legs. Each region is scored based on severity ranging from 0 (least severe) to 4 (most severe), provoking factors ranging from 1 (least severe) to 4 (most severe) for the mouth and jaw and from 0 (least severe) to 4 (most severe) for other regions. The maximum score for the motor severity scale is 120 and the score is calculated as the sum of each region’s scores (the individual region’s motor severity score = provoking factor × severity factor × weight). The disability scale has a maximum score of 30 and is calculated as the sum of each region’s scores, with the disability score for each region ranging from 0 (least severe) to 4 (most severe).

### Image acquisition

The MRI images were collected using a 3T MRI scanner (Tim Trio; Siemens, Erlangen, Germany). High-resolution 3D T1-weighted data were obtained using a magnetization-prepared rapid-acquisition gradient-echo pulse sequence with the following parameters: repetition time = 2530 ms, echo time = 4.45 ms, inversion time = 1100 ms, flip angle = 7^°^, matrix dimensions = 256 × 256, voxel size = 1 × 1 × 1 mm^3^, are 192 axial slices. DTI data were obtained using a single-shot echo-planar imaging sequence for 64 non-collinear directions with *b* = 1000 s/mm^2^ and one reference image without diffusion weighting with *b* = 0 s/mm^2^. Other main parameters included repetition time = 7000 ms, echo time = 91 ms, flip angle = 90^°^, matrix dimensions = 128 × 128, voxel size = 2 mm × 2 mm × 3 mm, field of view = 256 mm × 256 mm and 50 axial slices.

### Multidimensional structural analyses

**Figure 1:**
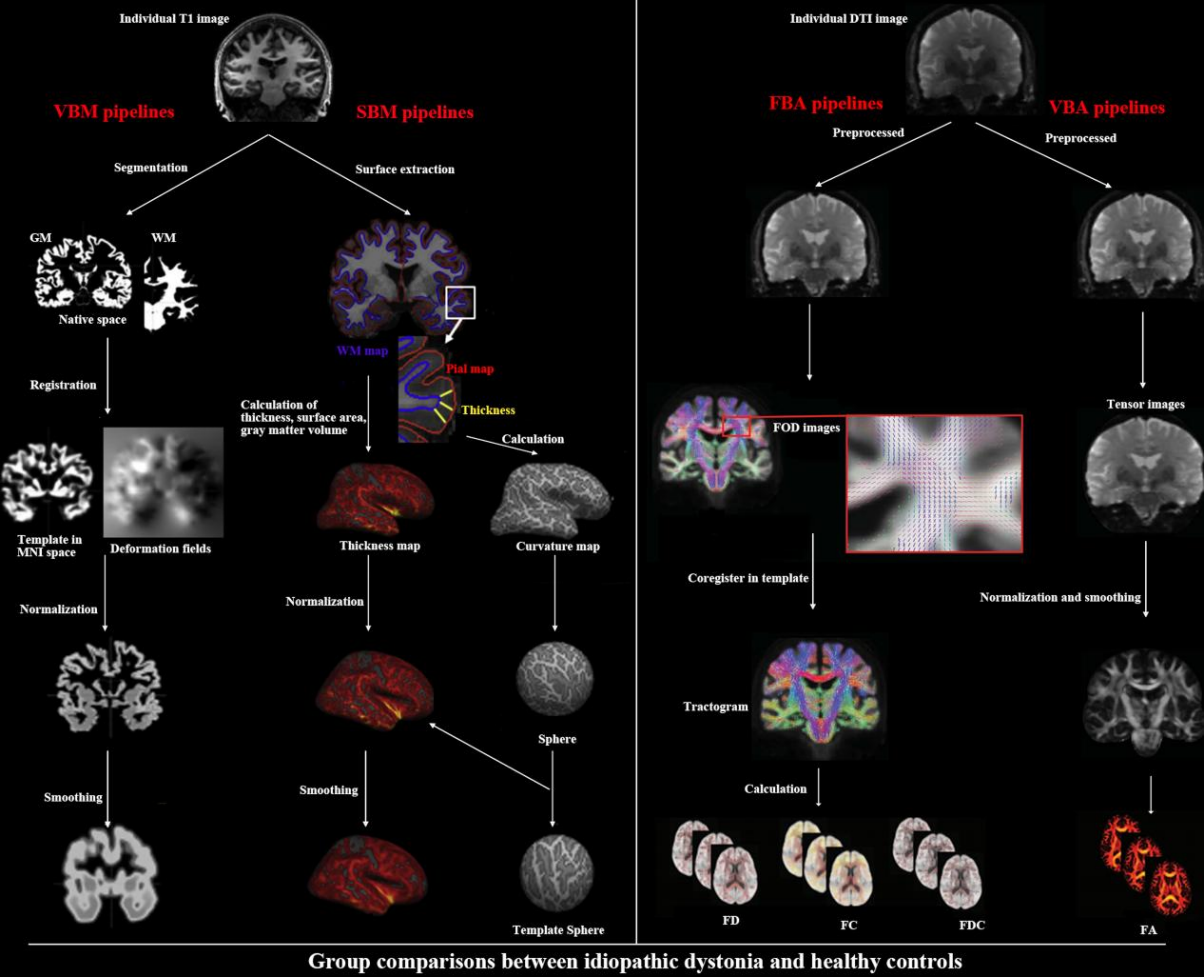
**Pipelines of multidimensional structural analyses.** Voxel-based morphology and surface-based morphology were performed for T1 images to investigate grey matter alterations, as well as the voxel-based analyses and fixel-based analyses were performed for diffusion tensor images to investigate white matter alterations. DTI, diffusion tensor imaging; FA, fractional anisotropy; FBA, fixel-based analysis; FC, fibre cross-section; FD, fibre density; FDC, fibre density and cross-section combined; FOD, fibre orientation distribution; GM, grey matter; MNI, Montreal Neurological Institute; SBM, surface-based morphometry; VBA, voxel-based analysis; VBM, voxel-based morphometry; WM, white matter.

Whole-brain VBM combined with SBM and whole-brain voxel-based analyses (VBA) combined with FBA were performed to investigate alterations in the grey and white matter, respectively (Fig. 1).

### Voxel-based morphometry

The T1-weighted images of all participants were preprocessed using the Data Processing and Analysis of Brain Imaging Toolbox (http://rfmri.org/dpabi). Initially, the quality of each image was visually checked, and no participant was excluded because of poor imaging quality. T1 images were processed using a one-step standard pipeline: the New-segment and Diffeomorphic Anatomical Registration Through Exponentiated Lie Algebra. All images were segmented into three tissues, grey matter, white matter and cerebrospinal fluid, and then transformed into a standard Montreal Neurological Institute space. Subsequently, these images were modulated to preserve regional volume information. Finally, the modulated grey matter images were smoothed with an 8-mm full width at half maximum for subsequent morphological analyses.

### Surface-based morphometry

Cortical surfaces were processed using FreeSurfer (http://surfer.nmr.mgh.harvard.edu/) with standard preprocessing pipelines. Briefly, image processing included skull stripping, automated Talairach transformations, segmentation of the subcortical white matter and deep grey matter structures, intensity normalization, tessellation of the boundary between the grey matter and white matter, automated topology correction and surface deformation along intensity gradients for the optimal placement of the borders between the grey matter, white matter and cerebrospinal fluid. In case of inaccuracies, manual editing was performed according to the FreeSurfer editing manual (https://surfer.nmr.mgh.harvard.edu/fswiki/FreeviewGuide/FreeviewWorkingWithData/FreeviewEditingaRecon), either by adding control points to help FreeSurfer identify white matter voxels or by removing the skull and dura if they were considered to be parts of the brain.

The CT, SA, grey matter volume (GMV) for cortical structures and estimated total intracranial volume (eTIV) were the outputs of FreeSurfer with its standard processing pipeline. The CT, SA and GMV of the left and right hemispheres were smoothed with a circularly symmetric Gaussian kernel of 5 mm full width at half maximum to improve the signal-to-noise ratio and further ensure a normal distribution of the results.

The GMV for the subcortical regions (bilateral thalamus, putamen, caudate, pallidum, hippocampus and amygdala) was automatically calculated using FreeSurfer (https://freesurfer.net/fswiki/SubcorticalSegmentation) during the –attorecon2 stage with its standard processing pipeline. This automatic subcortical segmentation is based upon the existence of an atlas containing probabilistic information on the location of structures automatically estimated from a manually labelled training set.^28^ During this step, each voxel in the normalized brain volume was assigned one of approximately 40 labels. Finally, the GMV was defined as the sum of all voxels in each label.

### Voxel-based analyses

DTI images were visually inspected to discard images with artefacts and were preprocessed using FSL 6.0 (http://www.fmrib.ox.ac.uk/fsl). Eddy current-induced distortions and head motion were corrected by aligning the scans to the b0 images. Subsequently, a binary brain mask was generated using the Brain Extraction Tool. The FA images were analysed using Brain FMRIB’s Diffusion Toolbox.

The VBA was performed using a statistical parametric mapping package (SPM 12; http://www.fil.ion.ucl.ac.uk/spm). The b0 image was standardized to the Montreal Neurological Institute space using the tissue probability maps within SPM 12 to estimate normalization parameters, which were then applied to all FA maps and resampled to 2 × 2 × 2 mm^3^. Finally, the normalized maps were smoothed using an isotropic Gaussian filter (8-mm full width at half maximum).

### Fixel-based analysis

We performed FBA using a pipeline in MRtrix3 (https://www.mrtrix.org/) to investigate white matter fibre-specific differences between patients with idiopathic dystonia and healthy controls. The DWI data were first preprocessed, including eddy current corrections, removal of Gibbs artefacts and intensity normalization. Next, we computed the fibre orientation distribution for each participant and used this information to derive a study-specific unbiased template representing the population fibre orientation distribution. All single-subject FODs were then registered with the population template, which was used as the reference image for the main analyses. Subsequently, we computed a white matter template mask and segmented the fixels from the fibre orientation distribution template. Following this, we calculated the FD, FC and FDC. Finally, we performed connectivity-based smoothing and statistical inference with connectivity-based fixel enhancement using two million streamlines and default parameters (smoothing = 10-mm full width at half maximum, *C* = 0.5, *E* = 2 and *H* = 3).

### Structural changes between groups

The group differences in whole-brain GMV for VBM, CT, SA, GMV for SBM, FA for VBA, as well as FD, FC and FDC for FBA between patients with idiopathic dystonia and healthy controls were compared using a general linear model analysis with age, sex and eTIV (only for VBM and SBM) as nuisance factors. Family-wise error corrections for multiple comparisons were performed using nonparametric permutation testing with 5000 permutations. The significance threshold was set at *P*_corrected_ < 0.05. Group differences in the volume of subcortical regions and diffusion metrics in brain regions showing significant differences between the two groups were analysed using two-sample *t*-tests with age, sex and eTIV (only for VBM and SBM) as covariates and corrected by false discovery rate corrections with *P*_corrected_ < 0.05.

### Relationships between structural alterations

To further examine the spatial relationship between grey matter atrophy and white matter disruption, we performed probabilistic tractography of the bilateral thalamus in 137 healthy controls.

Before probabilistic tractography, seed regions in the native T1 space were co-registered to the individual native DTI space using an inverse linear transformation and nonlinear deformations. Moreover, we checked the registration accuracy of each seed region slice by slice in the coronal, axial and sagittal planes within the native DTI space. The whole-brain anatomical connectivity patterns of the bilateral thalamus were analysed using the FSL package. First, we used a multiple-fibre extension based on a previously published diffusion modelling approach to calculate the probability distributions for two fibre directions at each voxel in the seed regions. Second, probabilistic tractography was applied to estimate the connectivity probability by sampling 5000 streamline fibres per voxel in the seed regions. For each sampled fibre, we first drew a sample direction from the local direction distribution at the seed voxel. Then, we advanced a fixed distance of 0.5 mm along this direction to a new position, where we again drew a new sample direction from the local distribution. This propagation procedure continued until the brain surface was reached or the path looped back. Third, the path distribution estimates were binarized with a connection probabilistic value *P* < 0.002 (10 out of 5000 samples) to reduce the number of false-positive connections and then warped into the standard Montreal Neurological Institute space. Fourth, a population map of the bilateral thalamus was obtained by averaging the binarized maps across each group. Fifth, we set a threshold of 50% in the population map (i.e. displaying only the voxels present in at least 69 healthy subjects) to identify the main white matter pathway. Finally, the results of the VBA and FBA were overlapped on the population map. In addition, correlations between abnormal thalamic volume and abnormal FA\FDC were performed in patients with idiopathic dystonia, with the results corrected using FDR corrections with *P*_corrected_ < 0.05.

### Statistical analyses

Age and eTIV were compared between the two groups using two-sample *t*-tests. χ^2^ test was performed for sex between the two groups. Since disease duration and severity in patients with idiopathic dystonia were not normally distributed, spearman correlations were performed between abnormal thalamic volume\FA\FDC and disease duration and severity in patients with idiopathic dystonia. Statistical significance was set at *P* < 0.05. These analyses were performed using the Statistical Package for the Social Sciences (SPSS) version 25.0 (SPSS Inc., Chicago, IL).

## Results

### Demographic information and clinical characteristics

In the current study, 147 patients with idiopathic dystonia and 137 healthy controls were included. The detailed breakdown of the BFMDRS scores for each dystonia cohort and the data for each subcomponent of the scores are provided in Supplementary Table 2. There were no differences in age, sex, or eTIV between the two groups (Table 1).

### Voxel-based morphometry

No significant differences in the grey matter were found between patients with idiopathic dystonia and healthy controls.

### SBM and volume comparison in subcortical areas

**Figure 2:**
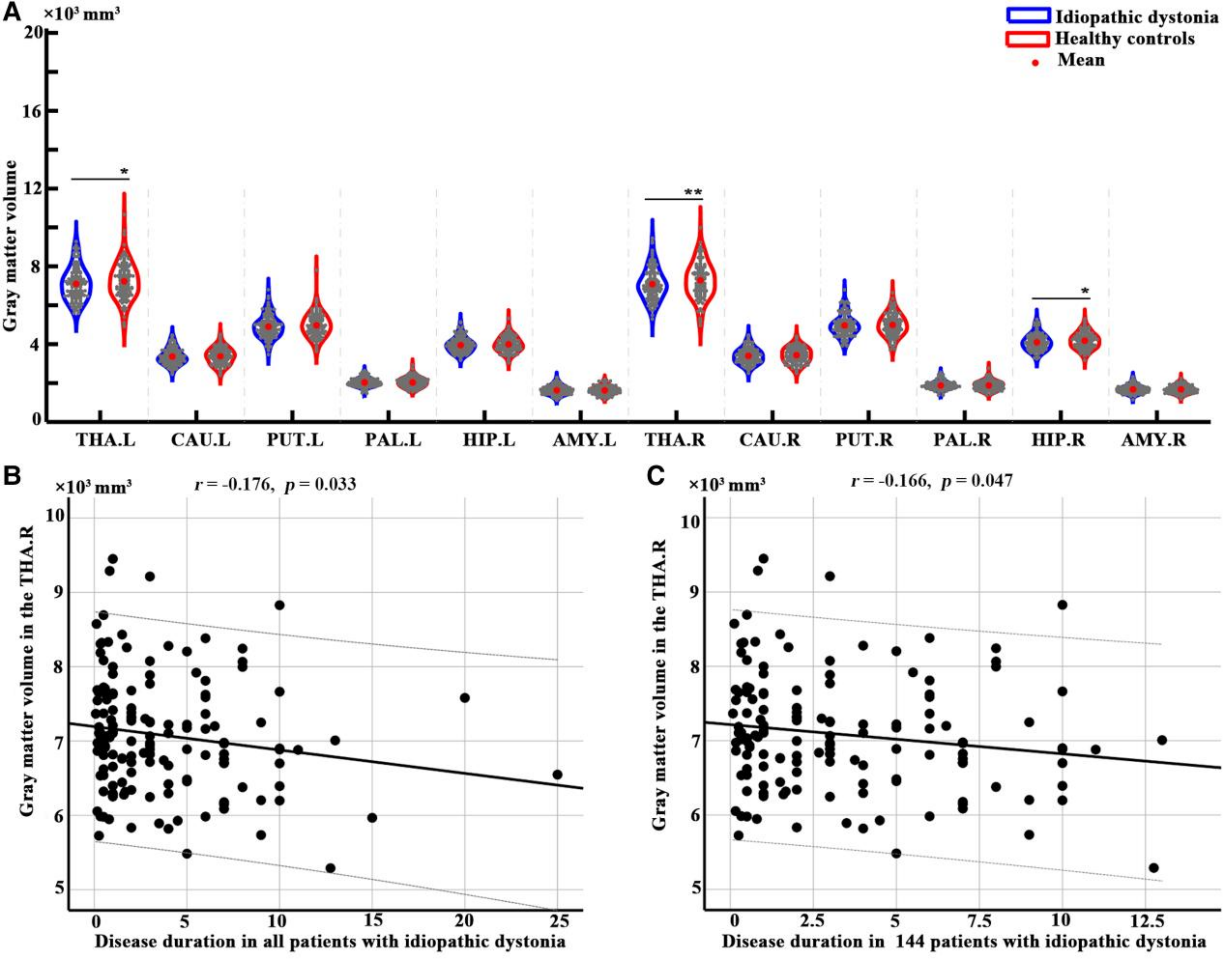
**Differences in volume in subcortical regions between 147 patients with idiopathic dystonia and 137 healthy controls.** (**A**) The results were obtained using two-sample *t*-tests with age, sex and estimated total intracranial volume as covariates. The grey dots within the violin plot represent the grey matter volume values for each subject in 12 different subcortical regions, and the central dots indicate the average grey matter volume for intra-group for the corresponding regions. The left side of the each violin plot represents patients with idiopathic dystonia group, and the right side of the each violin plot represents the healthy control group.* represents *P* < 0.05, ** represents *P* < 0.05, false discovery rate corrected. (**B**) Spearman correlations revealed that the decreased grey matter volume in the right thalamus was significantly correlated with disease duration in patients with idiopathic dystonia. (**C**) The decreased grey matter volume in the right thalamus was significantly correlated with disease duration in 144 patients with idiopathic dystonia (3 patients with disease duration over 15 years were excluded). Abbreviations of subcortical regions are listed in Table 2.

There were no significant differences in CT, SA, or GMV between patients with idiopathic dystonia and healthy controls. Group differences in the subcortical regions showed that the GMV of the right thalamus in patients with idiopathic dystonia (mean ± SD: 7078.587 ± 784.223) was significantly lower than in healthy controls (mean ± SD: 7277.587 ± 904.001) (*P*_corrected_ = 0.006). While the GMV of the left thalamus in patients with dystonia (mean ± SD: 7092.346 ± 816.061) showed a trend towards reduction compared with that in healthy controls (mean ± SD: 7224.300 ± 927.542) (*P*_uncorrected_ = 0.011), it did not reach statistical significance after correction for multiple comparisons. Moreover, decreased GMV in the right thalamus was correlated with disease duration, but not severity, in all 147 patients with idiopathic dystonia (*r* = −0.176, *P* = 0.033) and in 144 patients with idiopathic dystonia after excluding three outliers (*r* = −0.166, *P* = 0.047) (Fig. 2 and Table 2).

**Table 2:** Differences of grey matter volume in the subcortical regions between 147 patients with idiopathic dystonia and 137 healthy controls

Subcortical regions	Abbreviations	Idiopathic dystonia	Healthy controls	*P*	*P*_corrected_
**Left thalamus**	**THA.L**	**7092.346** ± **816.061**	**7224.300** ± **927.542**	**0**.**011**^a^	**0**.**069**
Left caudate	CAU.L	3364.141 ± 408.697	3371.685 ± 408.329	0.443	0.591
Left putamen	PUT.L	4903.878 ± 568.990	4964.709 ± 601.669	0.079	0.239
Left pallidum	PAL.L	2026.600 ± 210.983	2035.918 ± 244.171	0.328	0.492
Left hippocampus	HIP.L	3948.661 ± 376.930	3987.577 ± 383.869	0.106	0.255
Left amygdala	AMY.L	1630.332 ± 220.708	1625.427 ± 204.834	0.726	0.726
**Right thalamus**	**THA.R**	**7078.587** ± **784.223**	**7277.587** ± **904.001**	**< 0.001** ^b^	**0**.**006**
Right caudate	CAU.R	3391.753 ± 388.756	3423.754 ± 392.688	0.168	0.336
Right putamen	PUT.R	4958.975 ± 548.306	4985.593 ± 551.070	0.255	0.438
Right pallidum	PAL.R	1874.891 ± 206.496	1871.886 ± 232.575	0.674	0.726
**Right hippocampus**	**HIP.R**	**4101.290** ± **412.427**	**4168.655** ± **395.210**	**0**.**027**^a^	**0**.**111**
Right amygdala	AMY.R	1672.740 ± 199.563	1672.565 ± 194.923	0.528	0.634

Values shown as mean ± standard deviations. False discovery rate corrected.

^a^represents *P* < 0.05.

^b^represents *P* < 0.01 (results are shown in bold).

### Voxel-based analyses

**Figure 3:**
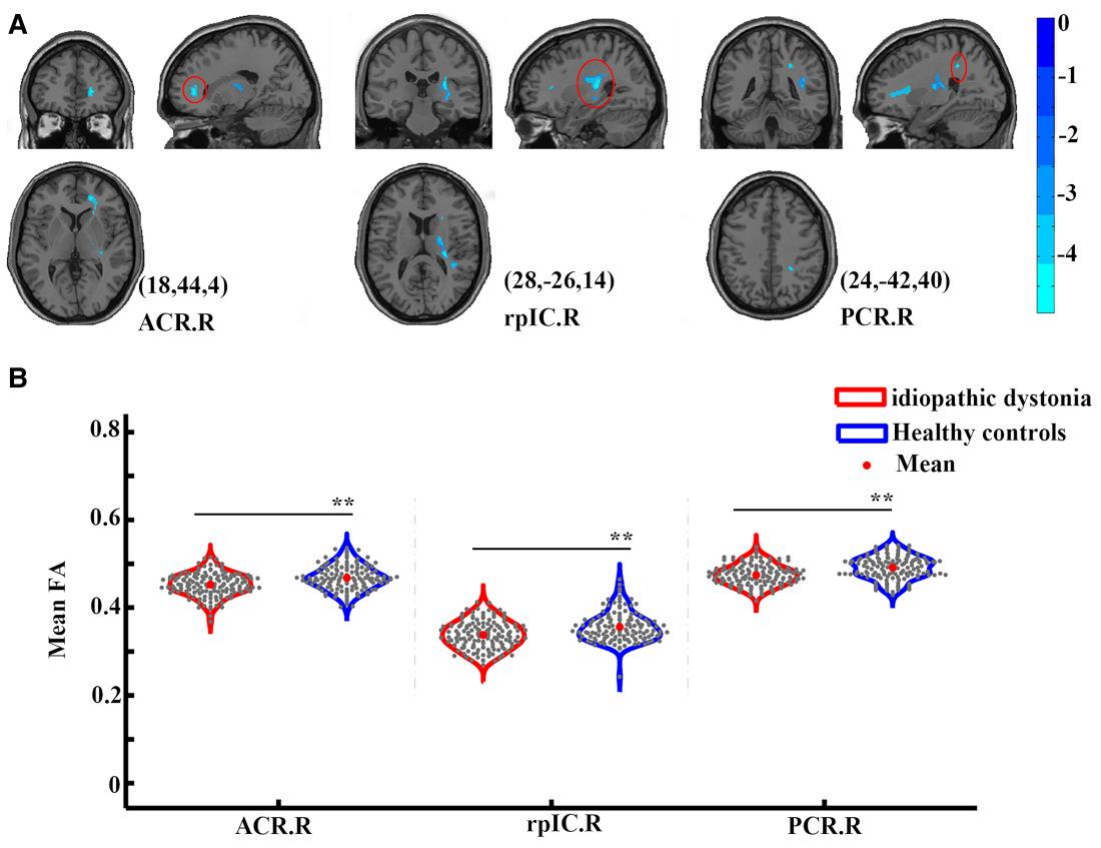
**Differences in white matter diffusion between 147 patients with idiopathic dystonia and 137 healthy controls.** (**A**) Decreased FA levels in three clusters were identified between groups via whole-brain voxel-based analysis. The results were obtained using a general linear model analysis with age and sex as covariates and corrected by family-wise error permutation testing of *P* < 0.05. Peaks with Montreal Neurological Institute coordinates (*x*, *y*, *z*) were shown for each cluster. The colour bar represents the *T*-value. (**B**) Differences in FA in regions obtained from whole-brain voxel-based analysis between groups. The results were obtained using two-sample *t*-tests with age and sex as covariates. The grey dots within the violin plot represent the mean FA values for each subject in three different brain regions, and the central dots indicate the average FA values for intra-group for the corresponding regions. The left side of the each violin plot represents patients with idiopathic dystonia group, while the right side of the each violin plot represents the healthy control group. ** represents *P* < 0.05 with false discovery rate corrections. ACR.R, right anterior corona radiate; FA, fractional anisotropy; L, left; PCR.R, right posterior corona radiate; R, right; rpIC.R, right retrolenticular part of internal capsule.

The FA values in the right anterior corona radiata (*P*_corrected_ < 0.001), right retrolenticular part of the internal capsule (*P*_corrected_ < 0.001) and right posterior corona radiata (*P*_corrected_ < 0.001) were significantly lower in patients with idiopathic dystonia compared with those in healthy controls (Fig. 3 and Supplementary Table 1). However, no significant correlations were observed among decreased FA levels, disease duration and disease severity in patients with idiopathic dystonia.

### Fixel-based analysis

**Figure 4:**
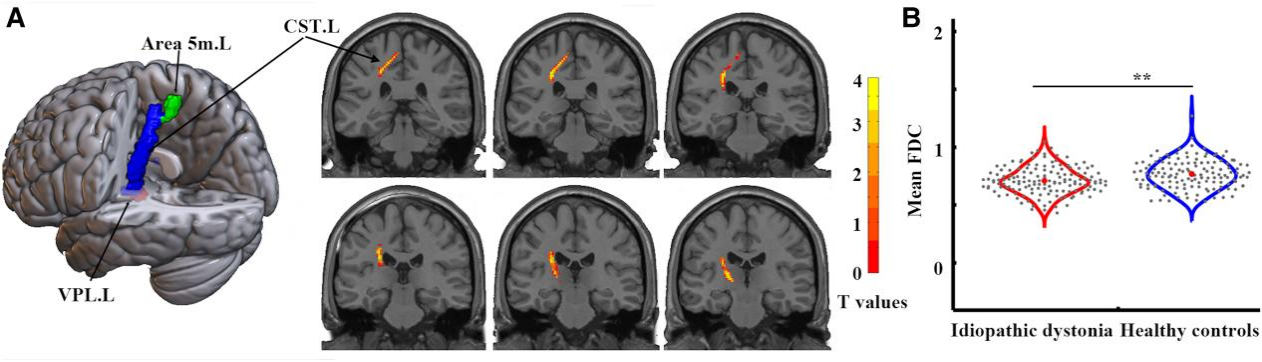
**Differences in fibre density and FDC between 147 patients with idiopathic dystonia and 137 healthy controls.** (**A**) Decreased FDC in the fibre tract connecting the left ventral posterolateral thalamic nuclear and left area 5 m was identified in patients with idiopathic dystonia compared with healthy controls. The results were obtained using a general linear model analysis with age and sex as covariates and were corrected for multiple comparisons with family-wise error permutation testing of *P* < 0.05. The left image was shown in MRIcroGL (https://www.nitrc.org/projects/mricrogl), and the right images were shown in MRICron (https://www.nitrc.org/projects/mricron). (**B**) Differences in FDC in regions obtained from whole-brain analysis between groups. The results were obtained using two-sample *t*-tests with age and sex as covariates. The grey dots within the violin plot represent the mean FDC values for each subject in the whole brain, and the central dots indicate the average FDC values for intra-group in the whole brain. The left side of the violin plot represents patients with idiopathic dystonia group, and the right side of the violin plot represents the healthy control group. ** represents *P* < 0.05, false discovery rate corrected. area 5 m. L, left area 5 m; CST.L, the left corticospinal tract; FDC, fibre density and cross-section combined; L, left; R, right; VPL.L, left ventral posterolateral nucleus.

Using the atlas of HCPex v1.1 (https://github.com/wayalan/HCPex), we found significantly decreased FDC in the fibre tract connecting the left ventral posterolateral thalamic nucleus and the left area 5 m in patients with idiopathic dystonia compared with healthy controls (*P*_corrected_ < 0.001) (Fig. 4 and Supplementary Table 1). However, no significant correlations were found between decreased FDC in this fibre tract and disease duration or severity in patients with idiopathic dystonia.

### Relationships between structural alterations

**Figure 5:**
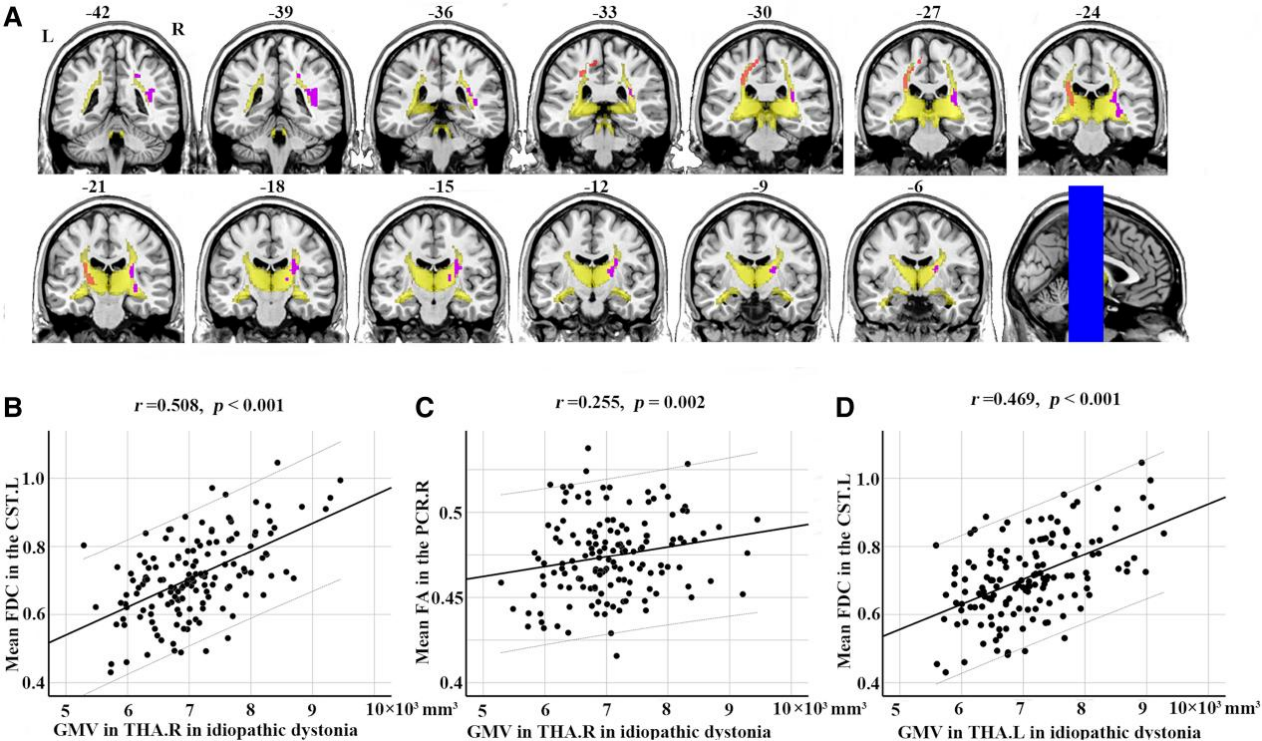
**Overlap results and correlations between morphological and microstructural alterations in idiopathic dystonia.** (**A**) Overlay of thalamocortical connections obtained from comparison results in FDC and FA between groups on the population maps of the probabilistic tractography patterns for bilateral thalamus in the healthy controls. The multi-slice views of fibres were shown with MRIcron (https://people.cas.sc.edu/rorden/mricron/). (**B**)-(**C**) The black dots of the scatter plot in figure B/C represent the relationship between the mean FDC in the CST.L or mean FA in the PCR.R and GMV in the THA.R in patients with idiopathic dystonia. Spearman correlations showed that GMV in the THA.R was significantly correlated with decreased FDC in the CST.L and FA in the PCR.R in 147 patients with idiopathic dystonia. (**D**) Spearman correlations showed that GMV in the THA.L was significantly correlated with decreased FDC in the CST.L in patients with idiopathic dystonia. CST.L, the left corticospinal tract; FA, fractional anisotropy; FDC, fibre density and cross-section combined; GMV, grey matter volume; L, left; PCR.R, right posterior corona radiate; R, right; THA.L, left thalamus; THA.R, right thalamus.

As shown in Fig. 5A, the right anterior corona radiata, right retrolenticular part of the internal capsule, right posterior corona radiata and fibre tract connecting the left ventral posterolateral thalamic nucleus and left area 5 m are components of the thalamocortical projections. The GMV in the right thalamus was significantly correlated with the mean FDC in the fibre tract connecting the left ventral posterolateral thalamic nucleus and the left area 5 m (*r* = 0.508, *P*_corrected_ < 0.001) and the mean FA in the right posterior corona radiata (*r* = 0.255, *P*_corrected_ = 0.002). In addition, the GMV in the left thalamus was significantly correlated with the mean FDC in the fibre tract connecting the left ventral posterolateral thalamic nucleus and the left area 5 m (*r* = 0.469, *P*_corrected_ < 0.001) in patients with idiopathic dystonia (Fig. 5B and C).

## Discussion

Using multidimensional structural analyses, we identified the structural changes in the bilateral thalamus and its connections to the cortex in patients with different forms of idiopathic dystonia. Additionally, we found that the decreased GMV in the thalamus not only correlated with disease duration but also with diffusion metrics in its connections to the cortex. However, the diffusion metrics in the abnormal thalamocortical projections were not correlated with disease duration in these patients. These findings suggest that structural changes in the thalamus and its connections to motor circuits exist in idiopathic dystonia. Moreover, microstructural disruption in thalamocortical projections might be secondary to thalamic atrophy (Supplementary Fig. 1).

Our findings regarding decreased GMV in the bilateral thalamus of patients with idiopathic dystonia are supported by many previous studies. Thalamic lesions can induce dystonia,^29^ suggesting an important role for the thalamus in the neuro-mechanisms of dystonia. Particularly, Marsden *et al*.^30^ concluded that the ‘abnormal input from the thalamus to the premotor cortex, due to lesions either of the thalamus itself or the striatum projecting by way of the globus pallidus to the thalamus’ may be causative in the pathophysiology of dystonia after reviewing 28 patients with focal or hemidystonia secondary to brain lesions. Structural neuroimaging studies have revealed decreased thalamic volume,^10^ as well as commonly changed FA and mean diffusivity in the white matter related to the thalamus^15,16,31^ in idiopathic dystonia. In addition, one study reported functional changes,^32^ and two reported white matter alterations^15,16^ in the thalamus of patients with blepharospasm-oromandibular dystonia. Functional MRI studies have shown increased cortical activation,^33,34^ glucose hyper-metabolism,^35,36^ decreased amplitude of low-frequency fluctuations,^37,38^ decreased functional connectivity profiles,^39,40^ as well as abnormal corticothalamic^41,42^ circuits in patients with idiopathic dystonia. However, almost all previous studies have reported no correlation between thalamic alterations and disease severity or duration in patients with idiopathic dystonia, making it difficult to determine whether thalamic atrophy is the pathological mechanism underlying idiopathic dystonia. Fortunately, using a relatively large sample size, we found that decreased GMV in the right thalamus significantly correlated with disease duration in idiopathic dystonia. These findings suggest that thalamic atrophy may be the pathological mechanism in idiopathic dystonia.

We identified decreased FA values in the right anterior corona radiata, right retrolenticular part of the internal capsule and right posterior corona radiata in patients with idiopathic dystonia. Consistent with our results, several previous studies have reported structural abnormalities in the anterior and posterior corona radiata. In addition, lower FA values were also identified in the right internal capsule in patients with spasmodic dysphonia,^43,44^ the dentatorubrothalamic tract in idiopathic cervical dystonia^45^ and thalamic pathway in idiopathic dystonia.^46^ Studies on blepharospasm have not identified FA differences^12^ but noted reduced average tract volumes and streamlined counts between the brainstem and motor cortex.^14^ Other studies involving larger cohorts of idiopathic dystonia have reported more mixed results, with some identifying higher FA while others found lower FA values.^47,48^ Using a study on dystonia obtained from UK Biobank, no significant FA values were observed between 76 patients with dystonia and 311 unaffected controls.^49^ However, these studies were mostly performed on small cohorts (less than 100), potentially contributing to poor statistical power. In line with our findings, a recent study using T1 images as features to build a model named DystoniaNet identified clusters in the anterior and posterior thalamic radiations as biomarker components, which included a large cohort of several forms of idiopathic dystonia.^50^

In addition to VBA, we also performed FBA to investigate and explore the morphometric properties of the fibre pathways in dystonia. These two methods have been performed in a previous study, which showed decreased FD in the right striatum, whereas no significant differences in the FA in cervical dystonia when compared with that in healthy controls.^51^ In the current study, we observed decreased FDC in the fibre tract connecting the ventral posterolateral thalamic nucleus and the area 5 m in patients with idiopathic dystonia compared with that in healthy controls. This fibre also goes through the striatum as seen in Fig. 5. Our results confirm previous findings and supplement further information. More consistent with our results, thalamocortical projection dysfunction has been reported in delayed-onset dystonia.^52^ The left ventral posterolateral thalamic nucleus corresponds to the ventral posterior nucleus of the thalamus, which relays sensory information from the second-order neurones of the neospinothalamic tract. Overall, our findings provide direct evidence that thalamocortical pathways are disrupted in different forms of idiopathic dystonia. These results are further supported by several previous studies, which reported a significant reduction in right (pre)motor- and left occipital-thalamic structural connectivity in dystonia,^16^ reduced integrity of cerebellothalamocortical fibre tracts in both clinically manifesting and non-manifesting *COL6A3* mutation carriers,^53^ decreased FA between the bilateral dentate nucleus and thalamus based on probabilistic tractography in patients with autosomal-recessive isolated dystonia (DYT27),^54^ and fewer thalamic prefrontal connections in patients with idiopathic dystonia.^46^

Although regions with differences in the FDC and FA could overlap in the fibres connecting to the thalamus, they are not exactly the same region. This discrepancy may be attributed to the use of varying methodologies. The FA is an index to measure axonal integrity, myelination, axon diameter and density, which cannot directly reflect a difference in the fibre properties.^55^ The FDC is a combination of FD and FC, particularly, the FD is used to address changes in white matter volume within a given voxel and the FC is used to address changes concerning the number of voxels that the entire fibre bundle occupied.^22^ Therefore, FDC could be used to estimate the total number of fibres in a fibre bundle. However, this method does not provide information on single neurones, but on fibre pathways, and its results should be rather interpreted as the ability of a fibre pathway to relay information. Besides our results, several previous studies that performed VBA and FBA simultaneously also showed discrepancies.^51,56,57^ To our knowledge, this is the first study to demonstrate a correlation between grey matter atrophy in the thalamus and disruption to the thalamocortical pathway in idiopathic dystonia. Although no significant correlations were found between decreased FA in the right anterior corona radiata, right retrolenticular part of the internal capsule, right posterior corona radiata and FDC in the fibre tract connecting the left ventral posterolateral thalamic nucleus and left area at 5 m, and disease duration in patients with idiopathic dystonia. Decreased FA in the right posterior corona radiata and decreased FDC in the fibre tract connecting the left ventral posterolateral thalamic nucleus and left area at 5 m were significantly correlated with decreased GMV in the right thalamus. In addition, decreased GMV in the right thalamus significantly correlated with disease duration in patients with idiopathic dystonia. Considering these findings collectively, we hypothesize that microstructural disruption of thalamocortical projections might be secondary to thalamic atrophy in idiopathic dystonia.

Despite these interesting results, certain limitations of this study must be addressed. First, patients with relatively limited forms of dystonia were recruited in our study, and it is necessary to include more types of dystonia to refine our findings. Second, as the onset rate is not the same among different subtypes of dystonia, we only recruited a relatively small sample size in blepharospasm-oromandibular dystonia and cervical dystonia. Moreover, the onset age is relatively lower in cervical dystonia than in other forms of dystonia. Thus, direct comparisons among these groups are not considered appropriate as the age and sample size could largely influence these results. Future studies should perform sub-group analyses with a larger number of patients with blepharospasm-oromandibular dystonia and cervical dystonia to assess the presence of a common pattern of change in different forms of dystonia. Furthermore, our DTI data were obtained using 64 non-collinear directions with *b* = 1000 s/mm^2^ and one reference image with *b* = 0 s/mm^2^, which yielded significant results. Similarly, several other FBA studies using a low number of gradient directions and/or low *b*-values have also yielded fairly encouraging results.^21^ However, high angular resolution diffusion imaging gradient schemes^58^ or multi-shell DTI data are highly warranted to verify our FBA results because the number of diffusion gradient directions and the b value have an impact on the overall qualitative aspects of white matter FODs. Finally, the correlation between GMV and white matter disruption was assessed using correlation analyses, but no direction was determined. Therefore, it is difficult to determine which brain region is affected first and which is secondary. Other direct ways of investigating effective connectivity should be explored in future studies.

In summary, our findings suggest that the thalamus and its projections are structurally impaired in idiopathic dystonia and that microstructural disruption in thalamocortical projections might be secondary to thalamic atrophy. Our findings will help researchers understand the pathophysiological mechanisms of dystonia better and facilitate the development of therapeutic strategies. In particular, the therapeutic modulation of network areas (e.g. the thalamus) using non-invasive or invasive stimulation technologies may improve dystonic symptoms.

## Supplementary Material

fcaf026_Supplementary_Data

## Data Availability

Data are available from the corresponding authors upon request. The Data Processing and Analysis of Brain Imaging Toolbox (http://rfmri.org/dpabi) was used to process T1-weighted images. FreeSurfer (http://surfer.nmr.mgh.harvard.edu) was used to process cortical surfaces. In case of inaccuracies, manual editing was performed according to the FreeSurfer editing manual (https://surfer.nmr.mgh.harvard.edu/fswiki/FreeviewGuide/FreeviewWorkingWithData/FreeviewEditingaRecon). FSL 6.0 (http://www.fmrib.ox.ac.uk/fsl) was used to process DTI images and analysed the whole-brain anatomical connectivity patterns of the bilateral thalamus. SPM 12 (http://www.fil.ion.ucl.ac.uk/spm) was used to perform VBA. MRtrix3 (https://www.mrtrix.org/) was used to perform FBA. The SPSS 25 (SPSS Inc., Chicago, IL, https://spss.en.softonic.com/) was used for statistical analyses.
