# Supplementary material for: Multidimensional structural analyses revealed a correlation between thalamic atrophy and white matter degeneration in idiopathic dystonia
Source: Brain Commun. 2025 Jan 20;7(1):fcaf026. doi: 10.1093/braincomms/fcaf026 (PMC11775609; doi:10.1093/braincomms/fcaf026)
Supplement: fcaf026_Supplementary_Data [file fcaf026_supplementary_data.pdf]

**SUPPLEMENTAL MATERIAL**

# Multidimensional structural analyses revealed a correlation between thalamic atrophy and white matter degeneration in idiopathic dystonia

## Supplemental figures

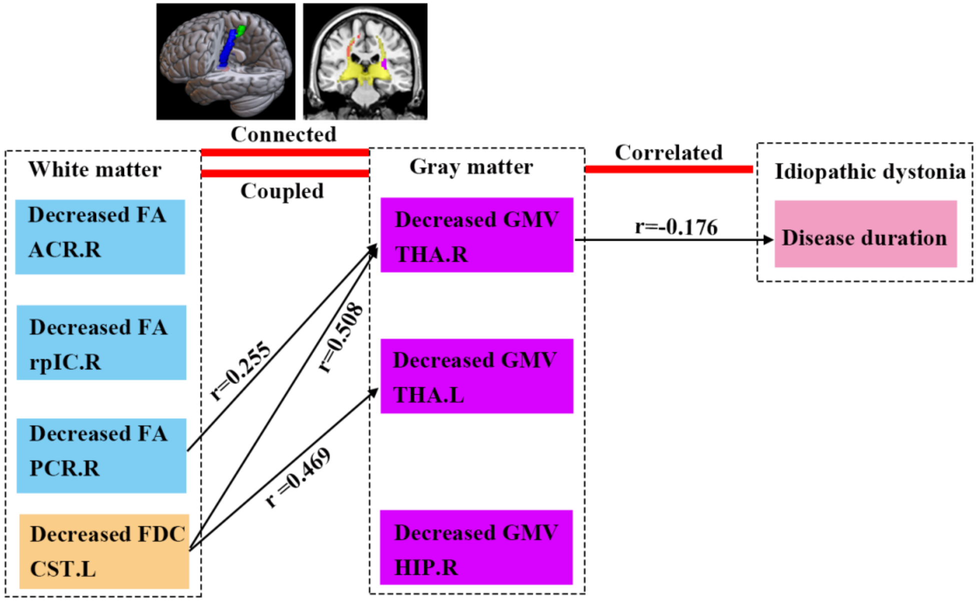


**Supplementary Figure 1. Summary of key findings between 147 patients with idiopathic dystonia and 137 healthy controls.** Spearman correlation analysis showed that the grey matter atrophy of thalamus is not only associated with white matter damage but also correlated with disease duration in patients with idiopathic dystonia. Abbreviations: ACR.R, right anterior corona radiate; FA, fractional anisotropy; FDC, fiber density and cross-section combined; CST.L, left corticospinal tract; GMV, gray matter volume; HIP.R, right hippocampus; L, left; PCR.R, right posterior corona radiate; R, right; rpIC.R, right retrolenticular part of internal capsule; THA.L, left thalamus; THA.R, right thalamus.

# Supplemental Tables

**Supplementary Table 1. Brain regions showing differences in fractional anisotropy and fiber density and cross section combined between groups.**

| Brain regions | Cluster size | MNI coordinates | Peak intensity | T-value | $P_{corrected}$ |
|---|---|---|---|---|---|
| **Fractional anisotropy** | | | | | |
| Right anterior corona radiata | 152 | 18, 44, 4 | -4.639 | 25.07 | < 0.001 |
| Right retrolenticular part of internal capsule | 452 | 28, -26, 14 | -4.946 | 35.48 | < 0.001 |
| Right posterior corona radiata | 15 | 24, -42, 40 | -4.138 | 18.659 | < 0.001 |
| **Fiber density and cross section combined** | | | | | |
| The fiber tract connecting the left ventral posterolateral thalamic nuclear and left area 5m | 262 | -20, -22, 10 | -3.871 | 23.667 | < 0.001 |

Abbreviations: MNI, Montreal Neurological Institute.

**Supplementary Table 2. The BFMDRS scores for each dystonia cohort.**

| | Blepharospasm (n = 74) | Blepharospasm-oromandibular dystonia (n = 31) | Cervical dystonia (n = 42) |
|---|---|---|---|
| Median BFMDRS-M subscores (range) | 7.5 (4.5-8) | 6 (3-14) | 6.75 (1.5-12.75) |
| Median BFMDRS-D subscores (range) | 0 (0-0) | 0 (0-2) | 1.5 (0-9) |

Abbreviations: BFMDRS-D/M = disability/motor section of the Burke-Fahn-Marsden Dystonia Rating Scale.
